# Digital health: The next evolution of healthcare delivery

**DOI:** 10.1097/01.NURSE.0000872464.40584.87

**Published:** 2022-09-22

**Authors:** Crystal A. Grys

**Affiliations:** **Crystal Grys** is a Team RN at Mayo Clinic Arizona in the Division of Community Internal Medicine in Scottsdale, Ariz., and an instructor of nursing at the Mayo Clinic College of Medicine and Science. She is enrolled in a DNP program at Duke University.

**Keywords:** accountable care organization, digital health, fee-for-service, healthcare delivery, patient-centered medical home

## Abstract

Healthcare delivery models have evolved from fee-for-service to incentivized care like patient-centered medical homes and accountable care organizations. This article discusses the evolution of healthcare delivery models and presents a vision for digital health.

**Figure:**
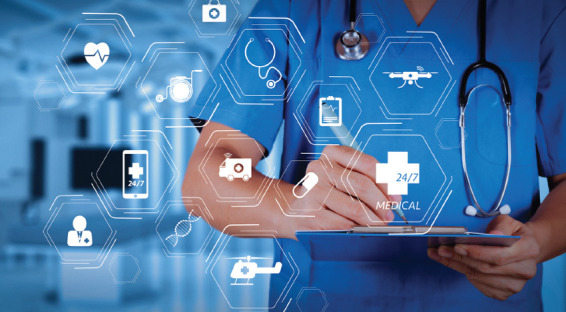
No caption available.

The state of healthcare in the US is analogous to world hunger. Many people are experiencing a striking lack of access to healthcare, while others have routine and facile access to high-value care.^1^ Like food, healthcare is not necessarily in short supply but suffers from a distribution problem and inefficient use.^2^,^3^ The COVID-19 pandemic has spurred innovation and accelerated the implementation of virtual care and telehealth. The adoption of more efficient healthcare delivery models is essential to solving healthcare's current distribution problem.

The term “Triple Aim” is often used to describe a framework to enhance the experience of caring for patients, improving the health of a population, and spending less money on healthcare.^4^ Recently, this term has been expanded to the “Quintuple Aim” with the additional goals of improving the well-being of the healthcare workforce and achieving healthcare equity.^1^,^5^ The healthcare delivery system of the US cannot be considered successful if it is not accessible and affordable to all demographics and populations within the country.

These five aims are not just related; they are intrinsically linked. In the US, it is estimated that about one-third of healthcare spending–roughly one trillion dollars–is wasted.^3^ This amount far surpasses the amount spent on the US military budget; however, overall health outcomes are not improving.^5^ The COVID-19 pandemic has further highlighted critical inequalities in American health outcomes.^6^ One possible cause is the longstanding, predominant model of Fee-For-Service (FFS). In response, patient-centered medical homes (PCMHs) and accountable care organizations (ACOs) have been developed to shift healthcare delivery toward greater value and better outcomes.

This article reviews common healthcare delivery models to provide context for the emerging model of Digital Health (DH). Nurses need to understand the evolution of these models and the potential changes to their practices so they can prepare to embrace and contribute to what may be the largest patient-care paradigm shift in their careers.

## Fee-For-Service: Outdated and inefficient

In the FFS model, a service must be performed for money to be earned. Unfortunately, this transaction is not a correlate of health. The natural outcome of this system is to keep finding ways to perform services, whether needed or not.^7^,^8^ The focus of the FFS delivery model was never on improving the health of the population or specific problems with individuals. Disturbingly, the FFS model disincentivizes resolving a healthcare problem.^7^

Consider a private practice with one exam room. The initial visit for a patient costs more than follow-up visits. Thus, a healthcare provider (HCP) will want to fill as much of their schedule as possible with initial visits. Follow-up visits are then delayed due to the prioritization of initial visits. In this system, the patient is not the focus, and achieving optimal health is rarely the goal since there is no reimbursement or payout for the patient achieving or maintaining health.^7^

The FFS model does not adequately function to improve the health of individuals or populations.^9^ New models of care delivery are required to fulfill the vision of the Quintuple Aim. Recent trends in healthcare delivery shift the focus to patient outcomes, engagement, and value, which are better models for increasing patient satisfaction and improving the health of a population.

## Shifting the care focus: Words, acronyms, and reality

The words and language used in healthcare are important as they often carry connotations and elicit an emotional response. For example, the PCMH, in which medical practice is designed to provide multidisciplinary or comprehensive primary care with cultural sensitivity, makes clear for whom the system is designed.^10^ Not only does “patient-centered” place the importance directly on the patient but using “home” implies comfort and wholeness. In PCMHs, reimbursements or payments are meant to address conditions, not pay for appointments. The intent is that health-related goals can be addressed quickly and efficiently, and with multiple modalities.^10^ Rather than being incentivized to see many patients, HCPs are incentivized to efficiently integrate and coordinate care to resolve health issues. The result can be a more efficient use of HCP time, allowing better access to care for the population. This is particularly true for populations with low health literacy, who are underserved, or whose occupation makes getting time off for appointments difficult.^9^

Another newer concept in care delivery is the ACO for those receiving Medicare. ACOs emerged as a care delivery model with the Affordable Care Act (ACA) and align costs, incentives, and outcomes. Healthcare organizations adopted the ACO model to meet cost and quality benchmarks in exchange for part of the savings derived from delivering a better quality of care.^11^ ACOs are supported by Medicare, Medicaid, and some commercial payors. The ACO model motivates a reverse-design approach, starting with the goal of quality care and building an efficient delivery model around that goal. The intent is to establish a system “perfectly designed” to get quality results. With ACOs, organizations facing declining reimbursement are offered a financial incentive to take the steps necessary to redesign their care systems.

Instead of competition and rising costs like the FFS model, ACOs encourage coordination and efficiency among HCPs, thereby eliminating redundant or perhaps unnecessary care.^13^

ACOs are most effective at driving collaboration across the network. When a patient subscribes to receiving care through an ACO, the HCPs within that organization agree to coordinate the patient's care. One study reported that the most consistent outcomes across payer types that utilized the ACO model were reduced inpatient use, reduced ED visits, and improved measures of preventive care and chronic disease management.^14^

## Impacts on quality and safety

The ACA includes some aspects of promoting outcomes and quality-driven practices to find new models to replace the failed FFS approach and ending Medicare reimbursement when a patient is readmitted to the hospital within 30 days of discharge.

For example, hospitals are penalized for episodes of care where patients are diagnosed with hospital-acquired conditions, including *Clostridium difficile* infection, catheter-associated urinary tract infection, and central line-associated bloodstream infection.^9^,^15^

These concepts represent the trend toward value and paying for desired outcomes. While promising, there is some risk of overlooking the centrality of the patient and their behavior. Not all patients are fully invested in their care or capable of contributing in helpful ways. Yet, a successful healthcare model must serve all patients. Otherwise, hospitals would be incentivized to provide care to informed and capable patients over those who are less skilled, have less access, or have a lower level of education. Practicing in such a way would impede health equity and increase health disparities. The latter also manifests in social determinants of health (SDOH)–factors inherent in a social environment and affect the health of a population in ways that are, at best, only indirectly related to their personal decisions. Examples of SDOH include safe housing; access to transportation; job opportunities; clean air and water; access to affordable nutritious food and physical activity opportunities.^16^,^17^ Healthcare models that account for SDOH are more practical, resilient, and successful.^18^

These trends of value, safety, and quality are affecting models of care delivery. Lawmakers have increased the list of hospital-acquired conditions that the Centers for Medicare and Medicaid Services penalize and incentivize positive outcomes through rebates or bonuses. Theoretically, these are good changes because they reward good outcomes and penalize bad outcomes; however, there are practical aspects of the penalties that can be difficult to manage. Consider situations whereby an infection is classified as hospital-acquired even when it probably was not. For this reason, bundled payments or Diagnosis Related Groups (that is, linking the case mix of the hospital to the resources consumed)^9^ are probably fairer than incentives and penalties because the former assigns a value to the care episode and allows the healthcare team to provide the most efficient care.

## Digital health

DH is a term that some have used to describe healthcare that is enabled or accessed by technology. This includes modalities like video or virtual visits and rapid and informal communication through electronic messaging.

Smartphones are often a conduit of information. A healthcare application can use a decision support tool to triage patient signs and symptoms, answer additional questions about or provide context for a disease or illness, patient education and home care advice, emergency precautions, and medication or testing. If automated, a system like this could offset the nonreimbursable administrative burden of busy internal medicine or primary care practices who respond to these types of inquiries in person via phone, online portal, or email messages. In return, triage and practice-based nurses would have more time to focus on billable, high-value activities like nurse visits.^19^ DH technology helped pave the way for advanced care at home and will continue to optimize those services.

Home-based testing could remove even more burden from the healthcare system. For instance, a patient with respiratory symptoms should not be coming to the HCP's office where they might expose others to a contagious pathogen. Instead, a mobile phone application might have a symptom-driven algorithm that screens for warning symptoms and can suggest that the patient perform a rapid nasal swab test at home for a pathogen like the influenza virus. If positive, the result would be sent to the patient's electronic health record and primary care provider. It would also present the patient with home care advice and automatically send a prescription to the patient's pharmacy of choice. The entire encounter could occur without the patient needing transportation, an appointment, or needing input from HCPs in some cases. Similarly, healthcare facilities could avoid being filled with appointments for patients with low-acuity symptoms. This would provide access for patients who have acute exacerbations or ongoing health concerns. All parties would benefit as the patients with respiratory symptoms get answers quickly, other patients maintain access to provider calendars, and HCPs spend their time on more complex issues and health maintenance.

One potential drawback of DH is access to technology. The digital divide is sufficiently important that digital technologies have been proposed as another determinant of health.^20^ Over the past 10 years in the US, smartphone adoption has increased from 35% to 85%, and the majority (61%) of Americans over 65 years old own a smartphone.^21^ To some, the vision of DH may seem impersonal, too automated, too invasive, or reinforce healthcare bias and inequality.^22^ Whether DH is a better approach than traditional measures should not be the focus; instead, the focus should be on whether digital healthcare can enable appropriate access to care for low-acuity situations while maintaining healthcare access for those with complex and chronic conditions.

To solve the problem of healthcare distribution, digital healthcare must be embraced. If leveraged appropriately, DH tools can maximize access to automated, trusted healthcare expertise and increase access to care for those who have historically been underserved. Providing digital healthcare with similar outcomes to in-person health should lower the cost of care and thus accomplish the goals of the Quintuple Aim. Nurses have opportunities to contribute to the optimization of DH. While some studies of nurse-led DH interventions have been undertaken, more work must be done.^23^

## Conclusion

Healthcare delivery is dynamic and seeks to fulfill the goals of the Quintuple Aim. The ACA facilitated new models of healthcare delivery, such as PCMH and ACOs. These models shifted the focus from the care provider to the patient. Future models will continue to strive to find the delicate balance of maintaining high-quality, low-cost, equitable care that engages and recognizes individual patients, improves the health of communities, and preserves the well-being of providers. DH modalities present a powerful opportunity for delivering care to individuals and positively impacting the health of communities.
